# Transforming growth factor activating kinase 1 regulates extracellular matrix degrading enzymes and pain-related molecule expression following tumor necrosis factor-α stimulation of synovial cells: an in vitro study

**DOI:** 10.1186/s12891-017-1648-4

**Published:** 2017-07-01

**Authors:** Kentaro Uchida, Shotaro Takano, Toshihide Matsumoto, Naoshige Nagura, Gen Inoue, Makoto Itakura, Masayuki Miyagi, Jun Aikawa, Dai Iwase, Atsushi Minatani, Hisako Fujimaki, Masashi Takaso

**Affiliations:** 1 Department of Orthopedic Surgery, Kitasato University School of Medicine, 1-15-1 Minami-ku, Kitasato, Sagamihara City, Kanagawa 252-0374 Japan; 2 Department of Pathology, Kitasato University School of Medicine, 1-15-1 Minami-ku, Kitasato, Sagamihara City, Kanagawa 252-0374 Japan; 3 Department of Biochemistry, Kitasato University School of Medicine, 1-15-1 Minami-ku, Kitasato, Sagamihara City, Kanagawa 252-0374 Japan

**Keywords:** Synovium, TGF-beta-activated kinase 1, Tumor necrosis factor-alpha, Matrix metalloproteinase 3, A disintegrin-like and metalloprotease (reprolysin type) with thrombospondin type 1 motif, 4, Cyclooxygenase-2, mPGES-1, Nerve growth factor

## Abstract

**Background:**

Recent studies have suggested that the tumor necrosis factor-α (TNF-α) pathway is a potential target for the management of osteoarthritis (OA). Transforming growth factor (TGF)-β-activated kinase 1 (TAK1) is essential in several cytokine-mediated cascades, including the TNF-α, interleukin-1 (IL-1), and TGF-β pathways. The role of TAK1 in synovial tissue in OA is not fully understood. Using synovial cells harvested from OA patients during surgery, we investigated whether TAK1 inhibition suppresses production of TNF-α-induced extracellular matrix degrading enzymes and expression of pain-related molecules.

**Methods:**

Synovial tissues were harvested from ten subjects with radiographic evidence of osteoarthritis (OA) during total knee arthroplasty. Synovial cells were cultured and stimulated with control (culture media), 10 ng/mL human recombinant TNF-α, or 10 ng/mL TNF-α and 10 μM of the TAK1 inhibitor (5Z)-7-oxozeaenol for 24 h. Real-time polymerase chain reaction (PCR) analysis was used to monitor expression of mRNA of the extracellular matrix degrading enzymes matrix metalloproteinase-3 (MMP-3) and a disintegrin-like and metalloprotease (reprolysin type) with thrombospondin type 1 motif, 4 (ADAMTS-4); and of the pain-related molecules cyclooxygenase-2 (COX-2), microsomal prostaglandin E synthase-1 (mPGES-1), and nerve growth factor (NGF). MMP-3 and NGF protein concentrations in cell supernatant were measured by enzyme-linked immunosorbent assay (ELISA). COX-2, mPGES-1 and ADAMTS-4 protein expression was also evaluated by western blotting.

**Results:**

TNF-α stimulated increases in ADAMTS-4 and MMP3 mRNA (2.0-fold and 1.6-fold, respectively, *p* < 0.05) and protein expression (21.5-fold and 2.0-fold, respectively). Treatment with the TAK1 inihibitor (5Z)-7-oxozeaenol reduced ADAMTS-4 and MMP3 mRNA (0.5-fold and 0.6-fold, respectively) and protein expression (1.4-fold and 0.5-fold, respectively) in OA synovial cells. COX-2, mPGES-1 and NGF mRNA (11.2-fold, 3.1-fold and 2.7-fold, respectively) and protein expression (3.0-fold, 2.7-fold and 2.2-fold, respectively) were increased by TNF-α. (5Z)-7-oxozeaenol treatment reduced mPGES1 and NGF mRNA (1.5-fold and 0.8-fold, respectively) and protein (1.5-fold and 0.5-fold, respectively).

**Conclusion:**

TAK1 plays an important role in the regulation of TNF-α induced extracellular matrix degrading enzymes and pain-related molecule expression. TAK1 may be a potential target for therapeutic strategies aimed at preventing osteoarthritis progression and pain.

## Background

Osteoarthritis (OA), the most common joint disease in humans, is characterized by cartilage destruction. Its main symptoms are joint stiffness and pain with associated joint space narrowing. OA severely limits physical activity and greatly impairs quality of life. The identification of molecules that inhibit OA progression and relieve pain is key to OA treatment.

Synovial tissues produce inflammatory cytokines and contribute to OA pathogenesis [1–3]. Several recent studies have suggested that elevation of tumor necrosis factor alpha (TNF-α) in synovial tissue is linked to OA progression and pain [4–7]. TNF-α-induces expression of matrix metalloproteinase-3 (MMP-3) and a disintegrin-like and metalloprotease (reprolysin type) with thrombospondin type 1 motif, ADAMTS-4 (aggrecanase 1) by OA synovial fibroblasts in mice [7]. The anti-TNF antibody infliximab has been shown to slow the progression of OA [5]. TNF-α promotes nerve growth factor (NGF) expression and contributes to peripheral sensitization in OA mice [6]. In addition, anti-TNF drugs had marked benefits on pain and walking distance, as well as synovitis and joint effusion in a patient with inflammatory knee OA [4]. Synovial fluid levels of TNF-α have been positively correlated with pain score in knee OA [8]. These observations suggest that regulation of the TNF-α pathway in synovial tissues may be key to the management of OA progression and pain.

Transforming growth factor (TGF)-β-activated kinase 1 (TAK1) is a member of the mitogen-activated protein kinase (MAPK) family [9]. TAK1 is required in the transduction cascades of several cytokine-mediated innate immunity signals, including the TNF-α, interleukin-1 (IL-1) and TGF-β pathways [10–12]. Several studies have suggested that TAK1 is involved in expression of extracellular matrix-degrading enzymes and pain-related molecules [13–18]. Specifically, TAK1 expression has been observed in the synovial tissues of OA and rheumatoid arthritis (RA) patients [14], and TAK1 knockdown in rheumatoid arthritis-affected synoviocytes reduced matrix metalloproteinase-3 (MMP-3) expression by IL-1β [15]. The selective TAK1 inhibitor (5Z)-7-oxozeaenol [19] reduced MMP13 and ADAMTS5 in human OA cartilage chondrocytes and synoviocytes without inflammatory cytokine stimulation while blocking degradation of human OA cartilage explants and progression of a rat OA model [18]; TAK1 inhibition reduced TAK1 activation in bovine synovial fibroblasts while increasing reactive oxygen species-induced cyclooxygenase-2 (COX-2) expression [16]; and TAK1 inhibition suppressed nerve growth factor (NGF) expression following TGF-β stimulation in cartilage of OA patients [13]. In addition, intra-articular injection of (5Z)-7-oxozeaenol reduced COX-2, MMP-3,-13, and ADAMTS4 expression in injured porcine cartilage [17]. However, the effect of TAK1 on F-F-α-induced extracellular matrix-degrading enzymes and pain-related molecules in human osteoarthritic synovial tissues remains undetermined.

We investigated whether TAK1 inhibition suppresses production of TNF-α-induced extracellular matrix degrading enzymes and expression of pain-related molecules.

## Methods

### Reagents

Human recombinant TNF-α was purchased from Biolegend (San Diego CA, USA) and (5Z)-7-oxozeaenol was purchased from Sigma (St. Louis, MO, USA).

### Patients

A total of ten participants with radiographic knee OA (unilateral Kellgren/Lawrence [K/L] grades 2–4) underwent total knee arthroplasty at our institution. The study included 3 men and 7 women aged 60–89 years (mean ± SD, 73.8 ± 8.3 years) with a mean ± SD body mass index (BMI) of 26.3 ± 2.6 kg/m^2^ (range 22.6–31.4). A sample of synovial tissue was harvested from the suprapatellar pouch of each operated knee during total knee arthroplasty surgery. Informed consent for participation in this study was obtained from each patient on the day before surgery.

### Immunohistochemistry

To determine the localization of phosphorylated TAK1 (p-TAK1) and TNF-α, the paraformaldehyde-fixed synovial tissue samples were embedded in paraffin and sliced into 3-μm thick sections. The sections were deparaffinized with xylene for 1 h, hydrated in serial dilutions of ethanol (100%, 95%, and 70%) and then rinsed in distilled water. For antigen retrieval, deparaffinized sections were immersed in 10 mM sodium citrate buffer pH 6.0 and maintained at 98 °C for 30 min. After cooling at room temperature, endogenous peroxidase was blocked with 3% hydrogen peroxide in methanol for 15 min. The slides were washed in phosphate-buffered saline (PBS) and incubated with 10% goat serum (Nichirei, Tokyo, Japan) at room temperature. Subsequently, the sections were incubated overnight at 4 °C with rabbit polyclonal primary antibody against p-TAK1 (Thr^184^/^187^) (cat.no. #4531, Cell Signaling Technology Japan, Tokyo, Japan). After washings twice in PBS, the sections were incubated for 10 min at room temperature with biotinylated anti-rabbit IgG (Nichirei). Subsequently, the sections were washed twice in PBS and incubated with horseradish peroxidase (HRP)-conjugated streptavidin for 5 min. Peroxidase activity was revealed by 3,3′-diaminobenzidine (Nichirei) and the sections were counterstained with Mayer’s hematoxylin.

### Synovial cell culture

Mononuclear cells were isolated from 500 mg synovium by digestion with 20 mL of 0.1% type I collagenase [20]. The cells were cultured (α-minimal essential media [MEM] + 10% fetal bovine serum) at 1 × 10^4^ cells/cm^2^ in six-well plates. The medium was changed twice over 7 days of culture. Subsequently, the cells were stimulated with control (culture medium), 10 ng/mL human recombinant TNF-α, or 10 ng/mL TNF-α and 10 μM (5Z)-7-oxozeaenol for 30 min or 24 h. The concentration of 10 μM (5Z)-7-oxozeaenol was determined based on previous studies [21, 22]. After 30 min, to monitor the efficiency of TAK1 inhibition, phosphorylation of p-38 MAPK was evaluated by western blotting. Subsequently, total protein and mRNA were extracted for real-time PCR and western blotting analysis and the culture supernatant was analyzed for NGF and MMP-3 by enzyme-linked immunosorbent assay (ELISA).

### Western blotting for p-38 MAPK phosphorylation

In vitro pharmacology of TAK1 inhibition by (5Z)-7-oxozeanol study shows that 3 or 10uM (5Z)-7-oxozeanol completely inhibits the phosphorylation of p-38 MAPK in the human B cell lymphoma cell line, DOHH-2 [19]. To monitor the efficiency of TAK1 inhibition in synovial cells, phosphorylation of p-38 MAPK was evaluated by western blotting. After 30 min of treatment, synovial cells were homogenized in sodium dodecyl sulfate (SDS) sample buffer. Proteins of the cellular homogenate (5 μg/lane) were separated by SDS–polyacrylamide gel electrophoresis and transferred electrophoretically to a polyvinylidene difluoride membrane in blotting buffer. The filter was blocked with 10% nonfat milk in 20 mM Tris, 137 mM NaCl, 0.1% Tween 20 [pH 7.6] (TBST) for 30 min at 20 °C and then incubated with rabbit polyclonal primary antibodies against phospho-p-38 MAPK (Thr^180^/Tyr^182^) (cat.no.#9211; Cell Signaling Technology Japan) or p38-MAPK (cat.no.#9212; Cell Signaling Technology Japan) for 1 h at room temperature. The membrane was incubated with peroxidase-labeled goat anti-rabbit IgG antibody (Zymed Laboratory, San Francisco CA, USA) for 1 h at room temperature. After washing, the immunoreactive bands were visualized by enhanced chemiluminescence (Super Signal; Pierce, Rockville IL, USA) and a luminescent image analyzer with an electronically cooled charge coupled device (CCD) camera system (LAS-4000mini; Fuji Photo Film Co., Tokyo, Japan).

### Real-time PCR analysis

Total RNA was isolated from the cultured synovial cells using an RNA extract reagent (TRIzol, Invitrogen, Carlsbad CA, USA) following the manufacturer’s protocol. The extracted RNA was used as the template for first-strand cDNA synthesis of MMP-3, a disintegrin and metalloproteinase with thrombospondin motifs-4 (ADAMTS-4), COX-2, microsomal prostaglandin E synthase-1(mPGES-1), and NGF using Moloney murine leukemia virus (M- MLV) reverse transcriptase (SuperScript III RT, Invitrogen) in reaction mixtures composed of 2 μL cDNA, 0.2 μM specific primer pair, 12.5 μL N′,N′-dimethyl-N-[4-[(E)-(3-methyl-1,3-benzothiazol-2-ylidene)methyl]-1-phenylquinolin-1-ium-2-yl]-N-propylpropane-1,3-diamine dye reagent (SYBR *Premix Ex Taq*, Takara, Kyoto, Japan), and nuclease-free water in a final volume of 25 μL. The primers were designed using Primer Blast open-access software (http://www.ncbi.nlm.nih.gov/tools/primer-blast/) and were synthesized at Hokkaido System Science Co., Ltd., Sapporo, Japan. The sequences of the PCR primer pairs are listed in Table 1. The specificity of the amplified products was examined by melt curve analysis. Quantitative PCR was performed using a Real-Time PCR Detection System (CFX-96; Bio-Rad, CA, USA) to determine relative mRNA expression levels. The PCR cycle parameters were as follows: 95 °C for 1 min, followed by 40 cycles at 95 °C for 5 s and at 60 °C for 30 s. mRNA expression was normalized to the levels of glyceraldehyde-3-phosphate dehydrogenase (GAPDH) mRNA.

**Table 1:** Sequences of the primers used in this study

Primer	Sequence (5′–3′)	Product size (bp)
MMP-3-F	GTGGAGTTCCTGACGTTGGT	164
MMP-3-R	TGGAGTCACCTCTTCCCAGA	
ADAMTS-4-F	AACACTGAGGACTGCCCAAC	159
ADAMTS-4-R	GGTGAGTTTGCACTGGTCCT	
COX-2-F	TGGCTGAGGGAACACAACAG	74
COX-2-R	AACAACTGCTCATCACCCCA	
mPGES-1-F	GGAGACCATCTACCCCTTCCT	81
mPGES-1-R	AAGTGCATCCAGGCGACAAA	
NGF-F	CCCATCCCATCTTCCACAGG	74
NGF-R	GGTGGTCTTATCCCCAACCC	
GAPDH-F	TGTTGCCATCAATGACCCCTT	202
GAPDH-R	CTCCACGACGTACTCAGCG	

### Enzyme-linked Immunosorbent assay

MMP-3 and NGF protein concentrations/100 μL of cell culture supernatant were determined using a human MMP-3 ELISA kit (R&D Systems, Inc., Minneapolis MN, USA) and an NGF ELISA kit (R&D Systems).

### Western blotting for ADAMT-4, COX-2, and mPGES1

To investigate ADAMTS-4, COX-2, and microsomal prostaglandin E2 synthase 1 (mPGES1) protein expression, cells harvested from five patients were stimulated with TNF-α, or 10 ng/mL TNF-α and 10 μM (5Z)-7-oxozeaenol, for 24 h. Using methodology described previously [16], synovial cells were then lysed in radioimmune precipitation (RIPA) buffer (Wako Pure Chemical Co., Inc., Osaka, Japan) supplemented with a protease inhibitor cocktail (Roche, Madison WI, USA). The protein concentration for each tissue extract was determined using the bicinchoninic acid (BCA) assay (Pierce, Rockford, Illinois, USA). Protein extracts (10 μg/lane) were separated by sodium dodecyl sulfate-polyacrylamide gel electrophoresis and were electrophoretically transferred onto polyvinyl difluoride membranes, which were then blocked with polyvinylidene fluoride (PVDF) blocking reagent (DS Pharma Biomedical, Suita, Japan) for 1 h. The blocked membranes were incubated overnight at 4 °C with rabbit polyclonal primary antibodies against ADAMTS-4 (cat.no.ab185722; Abcam), rabbit polyclonal antibodies against COX-2 (cat.no.ab52237; Abcam), or rabbit polyclonal antibodies against mPGES-1 (cat.no.ab62050; Abcam). The primary antibodies were diluted 1:1000 with a blocking reagent (ImmunoBlock, DS Pharma). The membranes were washed with phosphate-buffered saline containing 0.05% Tween and incubated with the secondary antibodies (GE Healthcare, Piscataway NJ, USA), which had been diluted 1:1000 with blocking reagent. Immunoreactive proteins were visualized by chemiluminescence using an ECL detection system (GE Healthcare) and exposing the membranes to x-ray film. Each band was quantified by densitometric scanning using the NIH software ImageJ. The densitometry readings of the bands were normalized to β-actin expression.

### Statistical analysis

Differences between the untreated and treated synovial cells were compared using one-way ANOVA with Fisher’s least significant difference test. A *p* < 0.05 was considered statistically significant. Statistical analyses were performed using commercial software (SPSS v. 19.0, SPSS, Chicago IL, USA).

## Results

### Localization of p-TAK1 in osteoarthritic synovium

**Fig. 1:**
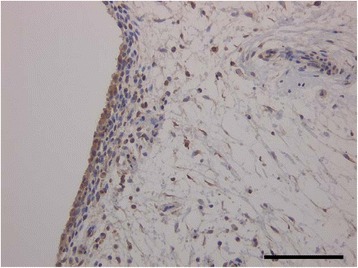
Immunolocalization of pTAK1 in synovium of OA patients. Immunolocalization of pTAK1. Scale bar =100 μm

To investigate the localization of p-TAK1, immunohistochemical analysis was performed. Immunoreactivity with p-TAK1 was observed in the synovial lining cells of OA patients (Fig. 1).

### Inhibition of p38 MAPK phosphorylation by (5Z)-7-oxozeaenol

**Fig. 2:**
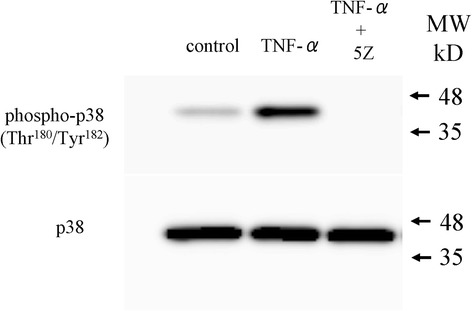
Effect of TNF-α and TAK1 inhibitor on p38 MAPK phosphorylation. Western blotting analysis for p38 MAPK phosphorylation. Synovial cells were stimulated with human recombinant TNF-α 10 ng/mL or 10 ng/mL TNF-α and 10 μM (5Z)-7-oxozeaenol (TNF-α + 5Z) for 30 min prior to protein extraction and analysis of p38 MAPK phosphorylation

Previous studies reported that TAK1 inhibition reduced phosphorylation of p38 MAPK [18, 19]. We confirmed that TNF-α stimulates p38 MAPK phosphorylation, which is completely inhibited by 10 μM (5Z)-7-oxozeaenol (Fig. 2).

### Effect of TAK1 inhibition on extracellular matrix degrading enzyme expression

Real-time PCR analysis revealed that the expression of ADAMTS-4 and MMP-3 increased significantly in synovial cells in the presence of exogenously added TNF-α (2.0-fold and 1.6-fold, respectively, *p* < 0.05, Table 2), and their expression was significantly reduced by the TAK 1 inhibitor (5Z)-7-oxozeaenol (0.5-fold and 0.6-fold, respectively, *p* < 0.05, Table 2).

**Table 2:** Effect of TNF-α and TAK1 inhibitor on MMP-3, ADAMTS-4, COX-2, mPGES1, and NGF mRNA expression

Gene	Fold increase			*p*		
	Control	TNF-α	TNF-α + 5Z	Control vs TNF-α	TNF-α vs TNF-α +5Z	Control vs TNF-α +5Z
ADAMTS-4	1.0 ± 0.1	2.0 ± 0.1^a^	0.5 ± 0.1^a,b^	0.0003	3 × 10^−4^	0.031
MMP-3	1.0 ± 0.3	1.6 ± 0.2^a^	0.6 ± 0.1^b^	0.018	0.028	0.096
COX-2	1.0 ± 0.3	11.2 ± 2.6^a^	4.5 ± 1.5^b^	0.007	0.014	0.065
mPGES1	1.0 ± 0.3	3.1 ± 0.5^a^	1.5 ± 0.5^b^	0.024	0.003	0.483
NGF	1.0 ± 0.1	2.7 ± 0.2^a^	0.8 ± 0.2^b^	0.006	0.003	0.312

Real-time polymerase chain reaction analysis for matrix metalloproteinase-3 (MMP-3), a disintegrin-like and metalloprotease (reprolysin type) with thrombospondin type 1 motif, 4 (ADAMTS-4), cycloxygenase-2 (COX-2), microsomal prostaglandin E synthase-1, and nerve growth factor (NGF) gene expression in synovial cell culture. Synovial cells were stimulated with human recombinant 10 ng/mL TNF-α (TNF-α), or 10 ng/ml TNF-α and 10 μM (5Z)-7-oxozeaenol (TNF-α + 5Z) for 24 h prior to the extraction and analysis of total RNA. All data are presented as the mean ± standard error (*n* = 6). ^a^ *p* < 0.05 compared with the untreated control. ^b^ *p* < 0.05 compared with the TNF-α

**Fig. 3:**
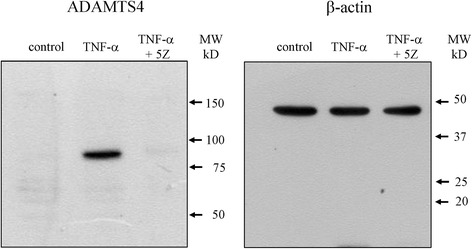
Effect of TNF-α and TAK1 inhibitor on ADAMTS-4 protein expression. Western blotting analysis for ADAMTS-4. Synovial cells were stimulated with human recombinant TNF-α 10 ng/mL or 10 ng/mL TNF-α and 10 μM (5Z)-7-oxozeaenol (TNF-α + 5Z) for 24 h prior to protein extraction and analysis of ADAMTS-4 protein

Western blotting analysis revealed that ADAMTS-4 increased in synovial cells in the presence of exogenously added TNF-α (21.5-fold) and that this expression was reduced by (5Z)-7-oxozeaenol (Fig. 3 and Table 3, *p* < 0.05). MMP-3 protein levels were also significantly increased in synovial cell culture supernatant in the presence of exogenously added TNF-α (2.0-fold) and this expression was reduced by (5Z)-7-oxozeaenol (*p* < 0.05, Table 3).

**Table 3:** Effect of TNF-α and TAK1 inhibitor on MMP-3, ADAMTS-4, COX-2, mPGES1, and NGF protein expression

Protein	Fold increase			*p*		
	Control	TNF-α	TNF-α + 5Z	Control vs TNF-α	TNF-α vs TNF-α +5Z	Control vs TNF-α +5Z
ADAMTS-4	1.0 ± 0.2	21.5 ± 2.8^a^	1.4 ± 0.3^b^	0.015	0.015	0.103
MMP-3	1.0 ± 0.2	2.0 ± 0.1^a^	0.5 ± 0.1^a,b^	0.001	1 × 10^−6^	0.015
COX-2	1.0 ± 0.3	3.0 ± 0.1^a^	2.2 ± 0.1^a^	0.001	0.052	0.022
mPGES1	1.0 ± 0.1	2.7 ± 0.1^a^	1.5 ± 0.1^a,b^	0.004	0.006	0.027
NGF	1.0 ± 0.2	2.2 ± 0.6^a^	0.5 ± 0.1^a,b^	0.032	0.017	0.009

Protein analysis for matrix metalloproteinase-3 (MMP-3), a disintegrin-like and metalloprotease (reprolysin type) with thrombospondin type 1 motif, 4 (ADAMTS-4), cycloxygenase-2 (COX-2), microsomal prostaglandin E synthase-1, and nerve growth factor (NGF) gene expression in synovial cell culture. Synovial cells were stimulated with human recombinant 10 ng/mL TNF-α (TNF-α), or 10 ng/ml TNF-α and 10 μM (5Z)-7-oxozeaenol (TNF-α + 5Z) for 24 h prior to the protein extraction. Expression of ADAMTS-4, COX-2, and mPGES1 proteins were analyzed by western blotting analysis. MMP3 and NGF protein levels in synovial cell culture supernatants were measured by ELISA. All data are presented as the mean ± standard error (*n* = 6). ^a^ *p* < 0.05 compared with the untreated control. ^b^ *p* < 0.05 compared with the TNF-α

### Effect of TAK1 inhibition on pain-related molecules

Similarly, COX-2, mPGES1, and NGF mRNA expression increased significantly in synovial cells in the presence of exogenously added TNF-α (11.2-fold, 3.1-fold and 2.7-fold, respectively) and were reduced by TNF-α with added (5Z)-7-oxozeaenol (Table 2).

**Fig. 4:**
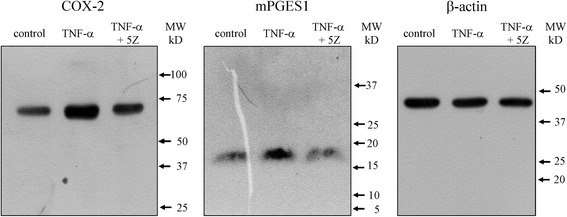
Effect of TNF-α and TAK1 inhibitor on COX-2 and mPGES1 protein expression. Western blotting analysis for COX-2 and mPGES1. Synovial cells were stimulated with human recombinant TNF-α 10 ng/mL or 10 ng/mL TNF-α and 10 μM (5Z)-7-oxozeaenol (TNF-α + 5Z) for 24 h prior to protein extraction and analysis of COX-2 and mPGES1 protein

Protein expression of COX-2 and mPGES1 also increased in synovial cells in the presence of exogenously added TNF-α (3.0-fold, 2.7-fold and 2.2-fold, respectively) and mPGES1 protein expression were significantly reduced by (5Z)-7-oxozeaenol (Fig. 4 and Table 3, *p* < 0.05). However, TNF-α-induced COX-2 protein elevation was not significantly reduced by (5Z)-7-oxozeaenol (*p* = 0.065). NGF concentration in cell culture supernatant was also significantly increased by TNF-α (2.7-fold; Table 3, *p* < 0.05) and decreased by added (5Z)-7-oxozeaenol (Table 3, *p* < 0.05).

## Discussion

This study showed that the phosphorylated TAK1 localized in synovial lining layer of OA patients. TAK1 inhibitor (5Z)-7-oxozeaenol reduced expression of the TNF-α-induced extracellular matrix degrading enzymes MMP-3 and ADAMTS-4 and the pain-related molecules mPGES-1 and NGF in the synovial tissues of OA patients.

MMP-3 and ADAMTS-4 have degradative effects on the extracellular matrix, and it has been suggested that they function as important inflammatory mediators in OA [7, 23–26]. In OA patients, plasma MMP-3 levels correlate closely with joint narrowing [24]. TNF-α stimulates MMP-3 and ADMAMTS-4 in synovial fibroblasts of OA mice [7]. It has been reported that TNF-α neutralization reduces ADAMTS-4 expression in synovial fibroblasts of OA patients [26]. In our study, TNF-α-induced MMP-3 and ADAMTS-4 expression was reduced by TAK1 inhibition with (5Z)-7-oxozeaenol in synovial fibroblasts, suggesting that TAK1 contributes to cartilage degradation by the TNF-α elevated MMP-3 and ADAMTS4 in OA synovium.

COX-2 and mPGES-1 enzymes are expressed in synovial lining cells. Prostaglandin E2 (PGE2) levels are elevated in inflamed synovial tissue [20, 27, 28] and TNF-α-stimulated synovial cells [29, 30]. PGE2 is considered to be the major contributor to inflammatory pain in arthritic conditions. Traditionally, non-steroidal anti-inflammatory drugs (NSAIDs), including selective COX-2 inhibitors, have been used to treat pain in OA. mPGES-1 is upregulated by inflammatory mediators, and gene deletion studies in mice indicate an important role for mPGES1 in inflammatory pain, revealing a different potential target for pain treatment in OA [31, 32]. We found that COX-2 and mPGES1 expression increased with exogenously added TNF-α and decreased with TAK1 inhibition, suggesting that TAK1 may be an important signaling initiator of the PGE2-signaling cascade stimulated by TNF-α in OA synovium.

TNF-α-upregulated-COX-2 and mPGES1 mRNA expression significantly decreased in the presence of (5Z)-7-oxozeaenol consistent with a previous report, where hydrogen peroxide-induced COX-2 elevation suppressed 5 μM (5Z)-7-oxozeaenol in bovine synovial fibroblasts. Although COX-2 protein expression was not completely suppressed, phosphorylation of p38 MAPK was reduced below baseline levels. Further investigations into the regulation of COX-2 by TAK1 in synovial cells are needed.

NGF also plays an important role in OA pain [6, 33–36], and the neutralization of NGF with tanezumab, an anti-NGF monoclonal antibody, has robust analgesic effects [33, 35, 36]. TNF-α stimulates NGF expression in OA synovial fibroblasts in vivo and in vitro [6, 37]. NGF and COX-2 expression have been considered to be regulated by inflammatory cytokines; however, several studies have reported that NGF and COX-2 are regulated by different pathways [38, 39]. COX-2 inhibitors have limited effect on IL1B-induced NGF expression in human synovial fibroblasts [38], but increase IL1B-induced NGF expression in human intervertebral disc cells [39]. Yazici et al. reported that combination therapy with tanezumab and NSAIDs was associated with greater improvement in knee and hip OA pain than NSAIDs alone [40]. In our study, TAK1 inhibition reduced not only mPGES1 expression but also NGF expression, suggesting that TAK1 may be therapeutic target for OA pain.

Several limitations of the present study warrant mention. First, we evaluated a single concentration of TNF-α at one time point. Second, this was an in vitro study. It remains to be determined if TAK1 inhibition will reduce OA progression and pain in vivo. Finally, we showed that TAK1 inhibition reduced MMP-3 and ADAMTS-4 levels, however, it remains to be determined whether blockage of TAK1 inhibits degradation of ECM proteins. This may be elucidated by investigating whether cell supernatants degrade purified extracellular matrix using an overlay assay [41].

## Conclusions

In conclusion, TAK1 upregulates TNF-α-induced extracellular matrix-degrading enzymes and pain-related molecule expression in OA synovial tissues. These properties suggest that TAK1 in synovial tissue may be a potential target for therapeutic strategies for osteoarthritic progression and pain.

## Abbreviations

ADAMTS-4: A disintegrin-like and metalloprotease (reprolysin type) with thrombospondin type 1 motif, 4
BCA: Bicinchoninic acid
COX-2: Cyclooxygenase-2
ELISA: Enzyme-linked immunosorbent assay
GAPDH: Glyceraldehyde-3-phosphate dehydrogenase
IL-1: Interleukin-1
IL-1β: Interleukin-1β
MAPK: Mitogen-activated protein kinase
MMP-3: Matrix metalloproteinase-3
mPGES-1: Microsomal prostaglandin E synthase-1
NGF: Nerve growth factor
NSAIDs: Nonsteroidal anti-inflammatory drugs
OA: Osteoarthritis
PCR: Polymerase chain reaction
PGE2: Prostaglandin E2
PVDF: Polyvinylidene fluoride
RIPA: Radioimmune precipitation
SYBR: N′,N′-dimethyl-N-[4-[(E)-(3-methyl-1,3-benzothiazol-2-ylidene)methyl]-1-phenylquinolin-1-ium-2-yl]-N-propylpropane-1,3-diamine
TAK1: Transforming growth factor (TGF)-β-activated kinase
TNFα: Tumor necrosis factor-α
α-MEM: α-minimal essential media
